# Frequency and Type of Thyroid Carcinoma in Patients With Multinodular Goiter

**DOI:** 10.7759/cureus.37921

**Published:** 2023-04-21

**Authors:** Anam Amin, Aalia Amjad, Ghazi Farman, Saad U Khaliq, Laraib Amin, Mahnoor Khan

**Affiliations:** 1 Department of Medicine, Northwest General Hospital and Research Center, Peshawar, PAK; 2 Department of Surgery, Khyber Teaching Hospital, Peshawar, PAK; 3 Department of Medicine, George Eliot Hospital, Nuneaton, GBR; 4 Department of Surgery, Northwest General Hospital and Research Center, Peshawar, PAK; 5 Department of Medicine, Northwest School of Medicine, Peshawar, PAK

**Keywords:** thyroidectomy, papillary carcinoma, multinodular goiter, malignancy, follicular carcinoma

## Abstract

Background

This study aimed to investigate the incidence and subtype of thyroid cancer in multinodular goitre (MNG) patients who underwent total thyroidectomy.

Methodology

A cross-sectional study was conducted at the Khyber Teaching Hospital, screening 207 MNG patients who received complete thyroidectomies between July and December 2022. The senior consultant diagnosed thyroid cancer based on a complete history, physical examination, and laboratory and radiological studies. Ultrasound-guided fine-needle aspiration cytology was performed by a senior consultant radiologist. Bethesda categories for all lesions were recorded. All patients underwent thyroidectomy, and the diagnosis of thyroid cancer was confirmed on histopathology.

Results

A total of 207 patients were included in the study, with a mean age of 45.55 ± 8.75 years. Out of 207 patients, 24 (11.59%) were diagnosed with thyroid cancer. Out of 62 male patients, 15 (7.25%) had thyroid cancer. Out of 145 female patients, only nine had cancer (p < 0.001). Nine patients with thyroid cancer had a body mass index (BMI) below 18, compared to only five patients with a BMI of more than 30 kg/m^2^. The difference in age distribution was not significant in our study (p = 0.102).

Conclusion

In conclusion, our study sheds light on the frequency and potential risk factors associated with thyroid cancer in patients with multinodular goiter. Our findings reveal that papillary thyroid carcinoma is the most commonly observed form of thyroid cancer in this patient population, with around 12 percent of patients diagnosed with thyroid cancer. Notably, our study highlights that male patients and those with a lower BMI may have a greater risk of developing thyroid cancer in the context of multinodular goiter. The findings of this study have important implications for the care and follow-up of MNG patients who receive total thyroidectomy. Further research is needed to investigate the type and prognosis of thyroid cancer in patients with MNG.

## Introduction

Multinodular goiter (MNG) is a common thyroid disorder characterized by the presence of multiple nodules in the thyroid gland. It is a benign condition, but a small proportion of patients with MNG may develop thyroid cancer. Patients with MNG have a five percent risk of developing thyroid cancer [1]. Thyroid carcinoma is one of the most frequent types of endocrinological cancers, and its occurrence has been on the rise globally [2]. Total thyroidectomy is regarded as the most effective surgical treatment for MNG and is also advised for patients with diagnosed thyroid cancer [3].

The clinical spectrum of thyroid cancer in association with MNG has been extensively studied. Several studies have revealed a greater incidence of thyroid cancer in MNG patients compared to those with a solitary thyroid lesion [4,5]. Nevertheless, thyroid cancer's incidence and prevalence vary according to the studied population, diagnostic techniques, and criteria for identifying thyroid cancer [6]. Thus, it is essential to research the prevalence and type of thyroid cancer in MNG patients undergoing total thyroidectomy in various populations and settings.

Some studies have suggested that the type of thyroid malignancy in individuals with MNG may be different from that in patients with solitary lesions. Those diagnosed with MNG have a greater risk of developing papillary thyroid cancer compared to patients diagnosed with isolated lesions. However, there is no significant difference in the occurrence of follicular thyroid carcinoma between the two separate groups [1,4]. However, other studies and comprehensive investigations have reported conflicting results [7]. In addition, the prognosis and treatment of thyroid cancer in patients with MNG may vary from those with solitary nodules [8].

This research is aimed at investigating the clinical presentation of thyroid cancer in patients with MNG admitted to the Khyber Teaching Hospital's tertiary care facility. This research project sought to study the incidence and subtype of thyroid cancer in MNG patients who received a complete thyroidectomy.

## Materials and methods

A cross-sectional study was undertaken by the Department of Surgery at Khyber Teaching Hospital between 1st July and 30th December 2022. All patients who reported to the outpatient department or were admitted through the emergency department for a complete thyroidectomy for multinodular goiter (MNG) with nodule sizes of 1.5 cm or greater were screened for malignancy. After obtaining ethical approval from the institutional review board (IRB) of Khyber Teaching Hospital (Reference # 213-21), patients were recruited using the convenience sampling technique, a non-probability technique.

The study included all adult patients with MNG, regardless of gender, and a normal thyroid profile (euthyroid). Excluded from the research were patients with congenital thyroid malformations or anomalies such as thyroglossal or branchial cysts, those who had undergone total thyroidectomy for previously detected malignant diseases, and those with a history of coagulation abnormalities. 

During the trial, 207 MNG patients received a complete thyroidectomy. Before data collection and surgery, all eligible patients were required to complete a written informed consent form. The senior consultant diagnosed thyroid cancer based on a complete history, physical examination, and laboratory and radiological studies, including ultrasound and computed tomography of the neck. To eliminate patients with euthyroid states, thyroid function tests were recommended, followed by ultrasound-guided fine-needle aspiration cytology (FNAC) of the thyroid gland to confirm the diagnosis of MNG. FNAC, under ultrasound guidance, was performed by an experienced consultant radiologist. The Bethesda category was recorded for each lesion, as summarized in Table 2. 

A total thyroidectomy was planned for patients with diagnosed MNG. Postoperatively, the thyroid specimen was delivered to the pathology department for confirmation of the malignancy. A predefined pro forma was utilized to enter patients’ sociodemographic and clinical characteristics, including age, gender, body mass index (BMI), and type of thyroid cancer. Data analysis was performed by IBM Corp. Released 2015. IBM SPSS Statistics for Windows, Version 23.0. Armonk, NY: IBM Corp. Quantitative variables such as age and BMI were subjected to mean and standard deviation calculations, while qualitative variables like gender and type of thyroid cancer were analyzed using frequency and percentage computations. The data were stratified for effect modifiers like age, gender, and BMI category. A post-stratification chi-square test analysis was performed to ascertain the distribution of thyroid cancer in different stratified groups. A p-value less than or equal to 0.05 was deemed statistically significant.

## Results

A total of 207 patients with a mean age of 45.55 ± 8.75 years were reported in the findings. We reported a female predominance in our study. Out of 207 patients, 24 (11.59%) were diagnosed with thyroid cancer (Table 1).

**Table 1:** Sociodemographic and clinical parameters of study population ASA: American Society of Anesthesiologists

Parameter	Mean or Frequency
Age in years	45.55 ± 7.80
Body Mass Index (BMI) in kg/m^2^	27.11 ± 3.23
Gender	
Female	145 (70.05%)
Male	62 (29.95%)
Frequency of ASA Class	
Class II	125 (60.39%)
Class II	54 (26.09%)
Class III	28 (13.53%)
Presence of Carcinoma on Histology	
No	183 (88.41%)
Yes	24 (11.59%)
Types of Thyroid Cancer	
Papillary	14 (58.33%)
Follicular	6 (25.00%)
Medullary	2 (8.33%)
Anaplastic	2 (8.33%)

It was found that 19 (9.2%) were Bethesda category VI (malignant), and 137 (66.2%) were Bethesda category II (benign) (Table 2). Out of the 19 Bethesda category VI tumors, 100% were diagnosed as malignant on histopathology. While one case in Bethesda category II was found to be malignant on histopathology. Out of the 12 cases in Bethesda category V, two were found to be malignant. Out of the 10 (4.8%) cases of Bethesda category IV and the 25 (12.1%) cases of Bethesda category III, one case in each category was found to be malignant on histopathology. 

**Table 2:** Distribution of Bethesda system for reporting thyroid cytopathology categories (N = 207)

Bethesda Category	N (%)
I (Non diagnostic)	4 (1.9%)
II (Benign)	137 (66.2%)
III (Atypia/follicular lesion of undertermined significance)	25 (12.1%)
IV (Suspected follicular neoplasm)	10 (4.8%)
V (Suspected malignancy)	12 (5.8%)
VI (Malignancy)	19 (9.2%)

The difference in age distribution was not significant in our study (p = 0.102). Out of 62 male patients, 15 (7.25%) had thyroid cancer. Out of 145 female patients, only nine had cancer (p < 0.001). Ten patients with thyroid cancer had a body mass index (BMI) of 18-25 kg/m2, compared to only five patients having a BMI of more than 39 18-25 kg/m2 (Table 3).

**Table 3:** Distribution of thyroid cancer with respect to different age groups, gender, body mass index, and ASA Class ASA: American Society of Anesthesiologists

Age Group	Carcinoma		p-value
	Yes (N = 24)	No (N = 183)	
18 – 40 year	11 (45.8%)	120 (65.6%)	0.102
> 40 year	13 (54.2%)	63 (34.4%)	
Gender			
				Male	15 (62.5%)	47 (22.71%)	< 0.001
Female	9 (37.5%)	136 (65.70%)					
Body Mass Index (BMI) in Kg/m^2^			
				18 – 25	9 (37.5%)	80 (25.7%)	< 0.001
26 – 30	10 (41.7%)	85 (46.5%)					
> 30	5 (20.8%)	18 (9.8%)					
ASA Class			
				Class I	7 (29.2%)	47 (25.68%)	0.750
Class II	15 (62.5%)	111 (60.66%)					
Class III	2 (8.3%)	25 (13.66%)					

## Discussion

Total thyroidectomy (TT) is widely practiced nowadays as the standard surgical treatment modality for the management of multinodular goiter (MNG) and thyroid malignancies [9]. The more conservative surgical alternatives have been steadily supplanted with TT, not only because the safety of thyroid surgery has increased but also because there is a risk of misdiagnosis or undetected thyroid cancer [10]. Moreover, if a redo surgery is at all required, there is an increased risk of complications [11].

Since previous studies had yielded widely disparate results regarding the frequency of malignancy in MNG, this study was conducted to gather local evidence to improve the management of these patients and to hail TT as the standard treatment option in MNG. The mean age of patients or subjects in our comprehensive study was 42.89 ± 8.75 years, which was comparable to the study done by Baloch et al., who reported a mean age of 42.38 ± 17.39 years in patients undergoing TT [12]. Similarly, another great researcher, Ghadhban et al., reported a mean age of 43.9 years [13]. Nevertheless, different studies by Kaliszewski et al. and Lin et al. reported higher mean ages of 53.3 ± 16.3 years and 52.4 ± 12.4 years, respectively [14,15]. Rageh et al. reported a younger mean age of 28.8 ± 10.2 years in their study [16].

Seventy percent of our patients were female. With p-values of 0.008 and 0.012, respectively, the incidence of malignancy and the frequency of distinct types of thyroid malignancies in patients with MNG receiving TT were shown to be statistically significant. Lin et al. (84.8%) and Baloch et al. (78.45%) similarly reported a higher proportion of female participants [15,17]. Ghadhban et al. (91.7%) and Ahmed et al. (94.5%) found even greater percentages of female patients [13,18]. Our findings were close to those of Imad et al., reporting an 11.2% incidence of cancer in MNG [19]. In the same study, the incidence of papillary carcinoma, follicular carcinoma, and squamous cell carcinoma was shown to be 66.7%, 22.2%, and 11.1%, respectively [19]. Ghadhban et al. reported an increased malignancy rate of 21.7% in cases with MNG, with papillary carcinoma accounting for 80% and follicular carcinoma accounting for 19.2% of the lesions [13]. According to another study by Nadeem et al., the incidence of cancer in MNG is 10.2% [20]. Thus, our findings were consistent with the published research.

There are certain limitations to our research. First, it is noteworthy that our study was exclusively conducted on individuals who were affiliated with a solitary clinic. This particular aspect of our research design may restrict the transferability of our conclusions to patients from other medical centers or diverse groups of individuals. Thus, the external validity of our findings may be somewhat limited due to the narrow scope of participants. Furthermore, it should be acknowledged that we did not delve into the analysis of the connection between specific characteristics of goiter and the probability of developing thyroid cancer. This research question could potentially be a focal point of further exploration in future studies, as it may provide valuable insights into the underlying mechanisms and risk factors associated with the development of this type of cancer. By conducting a more detailed examination of the relationship between goiter characteristics and the likelihood of developing thyroid cancer, we could potentially gain a deeper understanding of the various factors that contribute to the development and progression of this disease.

## Conclusions

Our research yielded some interesting insights into the frequency of and associated factors in patients with thyroid cancer. We concluded that 12% of the individuals with multinodular goiter (MNG) had thyroid cancer. Papillary thyroid carcinoma surfaced as the most common type in our cohort, with a predilection towards male patients. The initial results could have notable implications for managing and treating thyroid cancer. Highlighting the individuals who have an elevated likelihood of developing cancer could lead to early detection and intervention, potentially aiding in improved outcomes.
